# Exploration of teaching practice of analgesia and sedation in mainland China: CASER experience

**DOI:** 10.3389/fmed.2023.1010964

**Published:** 2023-02-08

**Authors:** Longxiang Su, Shu Li, Ran Lou, Ying Liu, Hua Zhang, Li Jiang

**Affiliations:** ^1^Department of Critical Care Medicine, Peking Union Medical College, Chinese Academy of Medical Sciences, State Key Laboratory of Complex Severe and Rare Diseases, Peking Union Medical College Hospital, Beijing, China; ^2^Department of Critical Care Medicine, Peking University People’s Hospital, Beijing, China; ^3^Department of Critical Care Medicine, Xuanwu Hospital Capital Medical University, Beijing, China; ^4^Department of Critical Care Medicine, Affiliated Hospital of Guizhou Medical University, Guiyang, Guizhou, China; ^5^Research Center of Clinical Epidemiology, Peking University Third Hospital, Beijing, China

**Keywords:** analgesia, sedation, assessment, training, China

## Abstract

**Objective:**

Analgesia and sedation assessments vary widely in clinical performance. This study investigated the cognition of intensivist and the importance of training for analgesia and sedation through the Chinese Analgesia and Sedation Education & Research (CASER) group training program.

**Methods:**

A total of 107 participants studied the training courses on the “Sedation, Analgesia and Consciousness Assessment of Critically Ill Patients” held by CASER from June 2020 to June 2021. Ninety-eight valid questionnaires were recovered. The content of the questionnaire included the preface, general information of the trainees, students’ awareness of the importance of analgesia and sedation evaluation and related guidelines, and professional test questions.

**Results:**

All respondents were senior professionals engaged in the ICU. A total of 92.86% believed that analgesia and sedation treatment were very important parts of the ICU, and 76.5% believed that they had mastered relevant professional knowledge. However, when evaluating the relevant professional theory and practice of the respondents from an objective point of view, it can be seen that only 28.57% of the respondents could reach the passing line in the specific case analysis scenario. Before participating in the training, 42.86% of the medical staff believed that analgesia and sedation treatment should be evaluated in the daily work of the ICU; after participating in the training, 62.24% of the medical staff believed that the evaluation was necessary and believed that they had improved after the training. Moreover, 69.4% of the respondents affirmed the necessity and significance of jointly undertaking the task of analgesia and sedation in Chinese ICUs.

**Conclusion:**

This study revealed that the assessment of analgesia and sedation is not standardized in the ICU in mainland China. The importance and significance of standardized training for analgesia and sedation are presented. The CASER working group thus established has a long way to go in its future work.

## Introduction

Analgesia and sedation have become very important and indispensable components of intensive care specialist treatment. The correct use of sedative and analgesic therapy can reduce the pain and fear of critically ill patients so that patients do not perceive, pay attention, remember or forget their pain during the severe stages and avoid anxiety, agitation and even delirium caused by the pain, which may improve patient condition and prognostic outcomes. Compared with Western countries, China’s analgesic and sedative treatment started late but has developed rapidly. In 2006, the Intensive Care Medicine Branch of the Chinese Medical Association released the first edition of the Chinese guidelines for analgesia and sedation for critically ill patients in 2006, and the guidelines were updated in 2018 (1). Following the update of the second edition of the Chinese guideline and the publication of the international PIADS guideline (2), domestic critical care colleagues have carried out different forms of study across the country, including self-study by physicians, department teaching, and promotion of related academic conferences. In the process of these studies, a common problem is exposed, and different readers are particularly prominent in the limitations of their own understanding of the guidelines: In actual clinical work, it was found that the theory and application are seriously disconnected. That is, many physicians have good theoretical basic knowledge, but they have subjective cognition and misjudgment about how to operate and implement related analgesia and sedation. To solve this outstanding clinical problem, the Critical Care Medicine Branch of the Chinese Medical Doctor Association established the China Analgesia and Sedation Education and Research (CASER) group and started special training in June 2020, aiming to improve theoretical understanding of analgesia and sedation. The supplement of clinical practice is helpful for the promotion and application of related concepts. Aiming at the contradiction between the significance and role of analgesia and sedation in critically ill patients and the clinical reality, this study investigated the cognition of bedside doctors and the importance of training for analgesia and sedation.

## Materials and methods

### Training group formation

The CASER group is entrusted by the Critical Care Medicine Branch of the Chinese Medical Doctor Association, with Professor LJ from the Department of Critical Care Medicine, Xuanwu Hospital, Capital Medical University as the project leader. The first batch of four lecturers was identified (YL, RL, SL, and LS). To ensure the consistency of training, the CASER group had conducted training and centralized learning with four fixed lecturers in advance. Before the formal college training of the trainees, three centralized lecturer trainings were organized beginning in March 2020. The training theme, specific training content, course structure organization and teaching methods were discussed and sorted out. After teacher training, the working group organized an assessment of the lecturers and confirmed the qualifications of the lecturers to ensure the homogeneity and consistency of the training.

### Investigation objects

From June 2020 to June 2021, the CASER group held training courses on “Sedation, Analgesia and Consciousness Assessment of Critically Ill Patients” in batches. The training course was open to recruiting students from all over the country, and the main groups were doctors and nurses working in the ICU. There were no more than 20 students enrolled in each training session, and registration automatically stopped when 20 students were reached. The main contents of the training were analgesia and analgesia assessment, sedation and sedation assessment, delirium and delirium assessment, and then there were related case simulations and discussions for a total of 10 cases. From February to March 2022, the working group distributed electronic questionnaires to all 107 trainees who participated in the training courses during the above mentioned year and collected them for analysis. The necessity of training was used as an indicator of sample size calculation. It was calculated that a sample of 81 patients would generate a 95% confidence interval estimate (CI), which is a range of likely values for the population proportion with precision (allowable error) of ±10% based on an estimated sample proportion of 70%. Given an anticipated dropout rate of 10%, the total sample size required was at least 90.

### Data collection and pilot testing

The questionnaire consisted of four parts (see Supplementary material): (1) Preface, which explained the purpose, significance, sponsoring institution and ethics-related matters of this research to the investigators. (2) General information of the trainees, gender, age, educational background, professional and technical title, work affiliation and medical unit and working years. (3) Students’ awareness of the importance of analgesia and sedation evaluation and related guidelines. (4) Professional test questions, including six theoretical questions about analgesia, sedation and consciousness assessment and six clinical case analysis questions, each with five points for a total of 30 points.

The questionnaire was organized and completed by the expert group according to the purpose of this research. The members of the expert group included five doctors from comprehensive ICUs in different provinces (with more than 15 years of work experience), two nurses and one medical statistician. The pilot testing of the questionnaire was completed by 10 trainees who participated in the training, and various data were collected, including the layout, structure, attractiveness, question setting, tolerance of the respondents, and understanding of the questions. SPSS software 20.0 was used for content validity analysis, and the Cronbach’s alpha value was 0.812, indicating that the questionnaire was effective.

## Results

### General characteristics

From June 2020 to June 2021, this project held 4 courses and trained a total of 107 trainees. After the questionnaires were distributed, a total of 98 valid questionnaires were returned. Among them, there were 51 males (52.04%) and 47 females (47.96%). Students aged 31–40 accounted for a maximum of 48 (48.98%). There were 86 Chinese doctors (87.76%) and 12 nurses (12.24%) among the trainees. Most of the academic qualifications were master’s degrees (56, 57.14%). Mainly with intermediate and senior titles, a total of 80 people (81.63%). Most of them worked in the comprehensive ICU of the university hospital (65.31%), and their working years were more than 10 years (58.16%) (Table 1).

**TABLE 1:** The general characteristics of the survey population.

Characteristic	Total (*n* = 98)
**Age range (years), *n* (%)**	
<25	2
25–30	16
31–40	48
41–50	28
>50	4
**Gender, n (%)**	
Male	51
Female	47
**Academic degree, *n* (%)**	
Associate’s degree	6
Bachelor’s degree	21
Master’s degree	56
Doctorate degree	15
**Profession, *n* (%)**	
Doctor	86
Nurse	12
**Level of professional title, *n* (%)**	
Junior	18
Intermediate	50
Senior	30
**Departments, *n* (%)**	
General ICU	64
Specialized ICU	30
Emergency department	2
Anesthesiology department	2
**Working years, *n* (%)**	
<5	15
5–10	26
11–15	33
16–20	11
>20	13
**Hospital type, *n* (%)**	
Tertiary hospital	93
Secondary hospital	3
Private hospital	2

### Cognition of physicians on the clinical assessment of sedative, analgesic, and delirium

**FIGURE 1:**
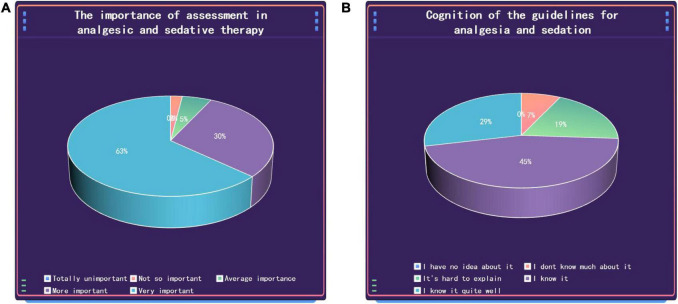
Medical staff’s understanding of the importance of sedation and analgesia assessment scales **(A)** and related guidelines **(B)**.

This study investigated the familiarity of clinicians with the Sedation and Analgesia Assessment Scale and related guidelines through subjective and objective questions. The results showed that the vast majority of medical workers believed that sedation and analgesia assessment was more important in the daily work of the ICU (*n* = 91, 92.86%). A total of 76.5% (*n* = 75) of medical workers believed that they were not satisfied with the PADIS guidelines and the Chinese guidelines for sedation and analgesia (Figure 1).

**FIGURE 2:**
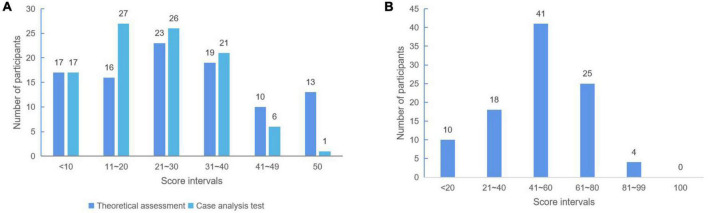
The distribution of scores of students’ test questions. **(A)** Theoretical assessment and case analysis test score. **(B)** The total score of the test.

To obtain an objective view of the familiarity of medical staff with the assessment of analgesia and sedation, medical staff were surveyed through a test questionnaire. Among them, the theoretical questions and clinical questions were out of 50 points each, with a total score of 100 points. The results showed that 42 people (42.86%) scored more than 30 points on theoretical questions, of which 13 people (13.27%) scored full marks. Twenty-eight (28.57%) of the case analysis questions scored more than 30 points, of which 0 were full marks. A total of 29 students (29.6%) had a total score of more than 60 points, and no student received a full score (Figure 2).

### The impact of training on the assessment of analgesia and sedation on the theory and practice of medical staff

**FIGURE 3:**
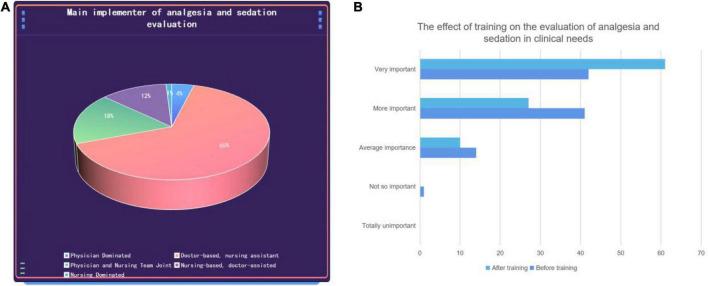
The impact of training on the assessment of analgesia and sedation on the theory and practice of medical staff. **(A)** Clinical practice of analgesia and sedation evaluation. **(B)** The effect of training on the doctor’s evaluation of analgesia and sedation before and after training.

The findings showed that training increased medical staff’s awareness of the importance of assessment. Before participating in the training, only 42 (42.86%) of medical staff believed that evaluation of analgesia and sedation treatment was necessary in the daily work of the ICU; after participating in the training, 61 (62.24%) medical staff believed that the evaluation was necessary. Our survey results show that 69.4% of medical staff believe that this work should be done by doctors or led by doctors. Among the 12 nurses who participated in the survey, no one believed that the work could be done independently by nurses, and seven nurses believed that doctors should be the lead and that the nurses should cooperate with them. A total of 57 think it can be done by doctors (Figure 3).

## Discussion

The study found that the vast majority of medical staff in Chinese ICUs have fully realized the importance of sedation and analgesia for the assessment and treatment of delirium, and the content of analgesia and sedation guidelines is well known. However, the specific content of the evaluation of sedation and analgesia for delirium treatment is generally familiar, and it cannot be used correctly in clinical practice. This is a problem that needs to be considered. In particular, the difference between China and foreign countries is that doctors are involved in more assessments of analgesia and sedation, which puts forward higher requirements for doctors and cooperation between doctors and nurses. Through our training, most doctors further improved their understanding of the importance of analgesia, sedation and delirium treatment evaluation, corrected some blind spots where they lacked understanding, and enriched their theoretical knowledge and clinical practice experience.

In addition to the primary disease, critically ill patients in the ICU also suffer from various painful feelings caused by the special environment of the ICU, treatment and nursing and other related operations and suffer from the double blow of the spiritual level, such as the lack of support from relatives around them and the fear of disease and death. Previous studies have shown that 50% of patients who have been in the ICU have painful memories of their experience in the ICU, and approximately 70% of patients had anxiety, restlessness and fear during their ICU stay (3–5). The Europain study even suggested that our routine movements, such as turning over and sucking sputum, can cause intolerable pain and adverse stimulation to patients (6). As ICU physicians have increasingly recognized the importance of improving the comfort of critically ill patients, analgesia, and sedation are a very important part of the basic treatment in ICU. The American Society of Critical Care Medicine published clinical practice guidelines for adult ICU patients as early as 1995 (7). This guideline was revised in 2002 (8), and the world-renowned PAD Guide was published in 2013 (9). In 2019, the PADIS guidelines were proposed again (2). The publication and update of a series of guidelines fully reflect the understanding of the latest concepts of analgesia and sedation treatment by intensive care physicians, pay more attention to the prevention and assessment of delirium, and further clarify the goal of ICU analgesia-based sedation and general sedation. A light sedation strategy enhances the management of delirium and emphasizes the important role of early activity and sleep. Analgesia and sedation is a subprofessional field, and systematic study and clinical practice are required for this related content to be more accurately grasped and to guide clinical treatment.

Intensive care medicine in mainland China has made great progress in the past 10 years. In particular, COVID-19 has brought some new improvements in the understanding of critical care and changes in clinical behavior. Analgesia and sedation are very important aspects (10). At present, most domestic intensive care physicians have fully realized the importance of analgesia and sedation, and clinical analgesia and sedation have become important treatment methods and means for critically ill patients (11). Through this study, it is believed that the low rate of the correct usage of the sedation and analgesia scale in clinical practice is due to the general familiarity with the assessment of sedation and analgesia treatment. There may be different understandings of the timing of analgesia and sedation, the duration of treatment, and the choice of drugs. The treatment of analgesia and sedation in mainland China is very different and heterogeneous. For example, some ICUs are treated with sedatives only, and some ICUs use deep sedation strategies that cause related complications. Throughout overseas surveys on analgesia and sedation, it was also found that different countries and regions also have very large differences in the choice and use of drugs (12–14). Therefore, the importance of consistent training, especially case-oriented training, and strengthening the application of the clinical application of analgesia and sedation assessment scales for medical staff has important practical significance.

As the saying goes, analgesia and sedation have no evaluation and no treatment. Therefore, the evaluation of analgesia and sedation has become a core issue. Our survey and study found that 75% of the survey respondents indicated that they understood the guidelines for analgesia and sedation, and 91.25% believed that the evaluation of analgesia and sedation was very important. However, the content of analgesia and sedation assessment was generally understood, and only 48.75% were able to achieve basic knowledge. Further examination of the doctor’s practical application ability through case analysis questions found that 22.5% of the people could reach 60 points, and only 1 person (1.25%) was able to judge all the differences in the survey correctly. This fully shows that practitioners have a clear understanding of the problem of analgesia and sedation, but the actual application is not ideal. If the evaluation cannot be accurately evaluated, then we cannot talk about the accuracy of the treatment. Therefore, in the evaluation of analgesia and sedation, relevant training needs to be strengthened, especially related operational practice, which is the cornerstone of analgesia and sedation treatment and the focus of this research.

Relevant training for medical staff was carried out in a targeted manner. After the training, 38.75% of medical staff needed to evaluate analgesia and sedation in their daily work in the ICU, and 60% of medical staff start related clinical practice. A total of 91.25% of the trainees believed that the training improved their awareness of the importance of analgesia and sedation evaluation and enriched their theoretical knowledge and clinical practice experience. In addition, more than 60% of Chinese surveyors for analgesia and sedation believed that the dominance lies with doctors, which is different from foreign nurse-led analgesia and sedation (2). Medical staff in China believe that it should be led by doctors and coordinated by nurses. Therefore, medical staff believe that cooperation between medical staff and nurses is essential in clinical work. Doctors provide feedback with nurses through bedside assessment and adjust strategies for care and treatment, which requires a deeper understanding of both doctors’ and nurses’ cognition of analgesia and sedation and is necessary to strengthen the popularization of relevant knowledge and understanding. The highlight of our training is the establishment of a group, first of all, a unified understanding of the lecturer team, the development of teaching materials, and an audience of doctors and nurses. The practice part is added to the training, especially through the workshop part. The real evaluation process can be seen, and it is no longer a rigid description in the textbook, which avoids misunderstandings. This lays the foundation for future training courses and systems.

In summary, we hope to improve in the following aspects. Firstly, a stable training core tutor team was established to regularly prepare lessons and study, and update new progress of analgesic and sedative treatment. Secondly, the practical courses based on theoretical basis, in addition to providing some standard teaching videos to display the evaluation method, are to build a simulated scenario for offline teaching and learning, or use more advanced virtual reality technology assistance. Third, establishing an assessment and evaluation system. In addition to the assessment of knowledge, we should investigate the form and content of teaching in order to improve the efficiency of teaching. Finally, a training textbook for Chinese national conditions is formed, combining analgesic and sedative teaching with the specific *status quo*, highlighting practicality, and analyzing common issues in targeted.

## Conclusion

Although the survey content is limited, it is not difficult to see from the data that Analgesia and sedation in China still need to strengthen training to unify professional understanding and achieve homogeneity in the management and treatment of analgesia and sedation in patients. The first step in training is to achieve an assessment of analgesia and sedation, which are key to laying the groundwork for subsequent treatment. CASER was established in compliance with this purpose. In the future, it will be committed to the promotion, application and research of knowledge of sedation and analgesia in China and will strengthen international exchanges and cooperation, as well as introduce the latest knowledge and concepts related to analgesia and sedation to China. Of course, more diversified teaching experiments and research will be carried out by CASER in the future.

## Data availability statement

The data analyzed in this study is subject to the following licenses/restrictions. The original data can be obtained from the corresponding author. Requests to access these datasets should be directed to LJ, casergroup@aliyun.com.

## Author contributions

LS and LJ conceived and designed this study. SL, RL, and YL organized this survey. HZ performed statistics. All authors contributed to the article and approved the submitted version.
