# Supplementary material for: Exploration of teaching practice of analgesia and sedation in mainland China: CASER experience
Source: Front Med (Lausanne). 2023 Feb 8;10:1010964. doi: 10.3389/fmed.2023.1010964 (PMC9945524; doi:10.3389/fmed.2023.1010964)
Supplement: Supplementary file 1 [file Table_1.DOCX]

**Investigation on training effect and demand of analgesia, sedation and consciousness evaluation**

Hello,

This questionnaire is designed to understand the teaching effect of the training workshop "Analgesia, sedation and awareness assessment" and the needs of trainers. This questionnaire is voluntary and anonymous, and all personal data involved in the questionnaire are confidential. It may take 10-15 minutes to complete this questionnaire. The questionnaire can only be completed once. Please preview before submitting.

Thank you for your support.

CASER Group

***Personnel information section***

1. **Your gender is**

A male

B female

1. **Your age is**

18-25

26-30

31-40

41-50

51-60

>60

**3. Your highest education is**

Undergraduate degree

Master degree

Doctor degree

**4. Your current position is**

Physician: junior intermediate senior

Nurse: junior, intermediate and senior

**5. Your department is**

Emergency Department

Comprehensive ICU

Specialized ICU: CCU RICU Extramental ICU Extracardiac ICU Comprehensive Internal Medicine ICU Comprehensive Surgery ICU Emergency ICU Trauma ICU

Others_____

**6. Your years of clinical work are**

<5 years

5-10 years

10-15 years

15-20 years

>20 years

**7. Your current work unit belongs to**

University Affiliated Third Class Hospital

Third Class Hospital

Second Class Hospital

First Class Hospital

Others______

***Training effect***

**1. The first choice of analgesic drugs in ICU:**

A benzodiazepines

B non steroidal

C opioids

D neuromuscular blockers

E central analgesics

**2. CPOT score does not include:**

A ventilator man-machine coordination

B patient's facial expression

C active limb movement

D passive limb movement

E state of consciousness

**3. Male, 29 years old, was transferred to ICU by emergency department after being injured by traffic accident 2 hours ago. The patient was restless, clenched the endotracheal tube, intermittently confronted with the ventilator, and became nervous when turning over. ECG monitoring showed: Bp 87/42mmHg, HR 112bpm, RR 33 times/min, SpO2 92%.**

**(1) The CPOT score of the patient is:**

A 3 points

B 4 points

C 5 points

D 6 points

**(2) In your opinion, which of the following analgesic treatment schemes should be selected first:**

A Morphine intramuscular injection

B Remifentanil continuous intravenous pumping

C benzodiazepines

D In order to observe the patient's mental state, analgesic treatment is not considered temporarily

**4. The following statements are correct**

A. Analgesia is the basis, and sedatives are adjusted according to the needs of the disease

B. Adopt individualized treatment plan to ensure the safety of cooperative treatment and medical care

C. Daily wake-up is not an absolute requirement

D. Multiple sedatives can be used together, and can be used in combination with analgesia or muscle relaxation

E. Comatose patients also need analgesia and sedation

**5. Among the following drugs, the most unsuitable sedative for patients with sinus bradycardia is**

A. Diazepam

B. Midazolam

C. Dextremetomidine

D. Propofol

6. Male, 64 years old, was admitted to the ICU through emergency treatment due to "4 hours after falling from a height". Head CT showed right frontal lobe hemorrhage, obvious cerebral edema, and left midline shift. The patient was restless, and human-computer confrontation was serious. After the analgesic treatment, the patient was slightly agitated, and the UAV antagonized. ECG monitoring showed that: Bp 184/95mmHg, HR 52bpm, RR 33 times/min, SpO2 94%. The RASS score of the patient after analgesia is:

**(1) The RASS score of the patient is:**

A -1

B 0

C 1

D 2

**(2) Which of the following sedative treatments should patients choose:**

A Continuous pumping of propofol

B Benzenediazepines continuously pumped

C Hibernating mixture

D patient is emotionally stable and does not need sedation

**7. In Glasgow score, when evaluating the physical activity**

A. Score both upper limbs separately

B. Score both lower limbs separately

C. Score the limbs separately, and take the limb with the best condition as the final score

D. Score the limbs separately, and take the worst limb as the final score

**8. Male, 21 years old, was sent to the emergency room by ambulance after falling from a building. Physical examination showed that he could not communicate, uttered vague sounds intermittently, could not open his eyes due to stinging pain, his upper limbs flexed, his lower limbs did not move, his lungs breathed loudly, his heart rhythm was uniform, and his lower limbs were positive for Pap's sign. The Glasgow score of the patient is**

A. 5

B. 6

C. 7

D. 8

**9. The following statement about CAM-ICU scoring is correct**

A. CAM-ICU score is not suitable for patients who are just awake after anesthesia due to the interference of anesthetic drugs

B. Patients who can cooperate quietly and communicate daily do not need CAM-ICU scoring

C. For patients who need sedation and analgesia, CAM-ICU score can be performed every day during the interval between sedation interruptions

D. Patients with unclear basic mental state cannot receive CAM-ICU scoring

**10. A 78 year old female patient was admitted to hospital due to respiratory failure. The patient was restless intermittently last night and could not sleep. During the ward round this morning, the patient cooperated quietly and told the doctor that he felt OK at present. During CAM-ICU evaluation, the number is wrong once, the answers to all questions are correct, and the instructions are wrong once. The following is correct**

A. Characteristic 1 positive, characteristic 2 negative, characteristic 3 positive, characteristic 4 positive, patient delirium

B. The patient is quiet and cooperative, without delirium

C. Characteristic 1 positive, characteristic 2 positive, characteristic 3 negative, characteristic 4 positive, patient delirium

D. Characteristic 1 positive, characteristic 2 negative, characteristic 3 negative, characteristic 4 positive, patient delirium

***Training needs***

**1. The importance of sedation, analgesia, consciousness assessment and treatment in your medical unit is**

0 (not important) to 10 (especially important) points using the scoring strip of the questionnaire star

**2. Before the training, your understanding of PADIS guidelines and guidelines for analgesia and sedation treatment in Chinese adult ICU**

A. Very familiar with

B. Quite familiar with

C. Understand part of it, but use less

D. Not very familiar with

E. Never heard of it

**3. Before attending the training, you feel the need for sedation, analgesia and consciousness assessment in your actual work**

A. Very necessary

B. Relatively necessary

C. Don't care

D. Not very necessary

E. Basically not necessary

**4. After attending the training, you feel the need for sedation, analgesia and consciousness assessment in your actual work**

A. Very necessary

B. Relatively necessary

C. Don't care

D. Not very necessary

E. Basically not necessary

**5. After attending the training, how do you think the importance of the medical team and nursing team in sedation, analgesia and consciousness assessment**

A. Completed by medical team

B. It should be led by the medical team and completed by the nursing team

C. The medical team and nursing team jointly complete

D. It should be led by the nursing team and completed by the medical team

E. To be completed by the nurse team

**6. Improvement in content and form of future courses: (multiple choices)**

A. Extend the time of theoretical courses □

B. Enrich the content of theoretical courses □

C. Strengthen workshop interaction □

D. Increase the frequency of training □

E. Training for medical group and nursing group respectively □

**7. In the training of "sedation, analgesia and evaluation of state of consciousness", you participated in**

A online training

B offline training (including theoretical teaching and actual case discussion)

**8. After attending this training, which of the following is more suitable for your situation:**

A Improved the level of relevant theoretical knowledge

B Improve the level of relevant clinical practice

C Improve the level of theory and clinical practice at the same time

D. The benefits are not obvious

**9. Which type of teaching do you prefer**

A. Better offline form effect

B. Better online form effect

C. The effect is quite good

D. The combination of the two is more effective
